# Role of gut microbiota in the pathogenesis of colorectal cancer; a review article 

**Published:** 2018

**Authors:** Somayeh Jahani-Sherafat, Masoud Alebouyeh, Sharareh Moghim, Hamed Ahmadi Amoli, Hajieh Ghasemian-Safaei

**Affiliations:** 1 *Microbiology Department, Faculty of Medicine, Isfahan University of Medical Sciences, Isfahan, Iran*; 2 *Foodborne and Waterborne Diseases Research Center, Research Institute for Gastroenterology and Liver Diseases, Shahid Beheshti University of Medical Sciences, Tehran, Iran *; 3 *Sina Hospital, Tehran University of Medical Sciences, Tehran, Iran*

**Keywords:** Gut microbiota, Pathogenesis, Colorectal cancer

## Abstract

Colorectal cancer (CRC) is one of the most frequently diagnosed cancers worldwide. Lifestyle is identified as one of the most important risk factors for CRC, especially in sporadic colorectal cancer. The natural composition of the gut microbiota changes rapidly during the first decade of life. Maintaining homeostasis in the gut is essential as structural and metabolic functions of the commensal microbiota inhibit gut colonization of pathogens. Dysbiosis, imbalance in function or structure of gut microbiota, has been associated with a variety of diseases, such as colorectal cancer. The aim of this review was to investigate the possible links between the dysbiosis in gut microbiota and colorectal cancer, and the potential role of anaerobic gut microbiota in the pathogenesis of colorectal cancer. Based on this review, various studies have shown that some of the gut microbiota such as anaerobic bacteria significantly increased in CRC patients, but we suggest more investigations are required to assess the importance of these bacteria and their metabolites in the pathogenesis of CRC are required.

## Introduction

  Colorectal cancer (CRC) is one of the top three most frequently diagnosed cancers worldwide, with nearly 1.4 million new cases diagnosed in 2012. More than 50 percent of colorectal cancer cases were reported in developed countries with the least incidence in Africa and Asia (1). Although developing countries are low-risk countries for CRC, particularly among the older population, but the age standardized rate within the young population in some Asian countries such as Iran and Pakistan, is as the same as US inhabitants (2,3). Despite extensive research exact etiology for CRC is still unknown, but genetic and environmental factors have been implicated as disease’ risk factors. The majority of CRC cases occur sporadically and less than 25 percent of CRC cases are hereditary (4). The similar incidence in young population between developed and developing countries seems associated with variations in lifestyle. Lifestyle is one of the most important risk factors for CRC, especially in sporadic colorectal cancers. Change in lifestyle factors, such as a diet rich in processed foods, animal fat and red meat with a low intake of fiber and fruits, decrease of physical inactivity and obesity, is thought to change the gut microbiota composition and increase the risk of disease in developing countries (5). 

 The colon is exposed to a large number of microorganisms. Approximately more than 10^13^ bacteria harbor in the adult human colon and other parts of the large intestine. During the first year of life, quick changes occur in the variety and composition of the microbiota. This composition, shaped by contact to environmental factors such as diet, antibiotic therapy, hospitalization, chemical exposure, and contact with the vaginal microbiota during birth, could be a connection between life style and accumulation of mutation in host (6-8). 

Gut microbiota are predominantly strict anaerobes, including *Bacteroides*, *Eubacterium*, *Bifidobacterium*, *Fusobacterium*, *Peptostreptococcus,* and *Atopobium*, while facultative anaerobes, such as *Enterococci*, *Lactobacilli*, *Enterobacteriaceae*, and *Streptococci,* form a minor portion of inhabitants (9). Maintaining this structure is essential for the gut hemostasis because structural and metabolic functions of the commensal microbiota inhibit gut colonization of pathogens. Microbiota participate in the production of short chain fatty acids and proteolytic fermentation by fermentation of anaerobic carbohydrate. Short chain fatty acids elaborate butyrate, propionate and acetate, used as a source of energy in gut and helps to proliferation and differentiation of intestinal epithelial cells (10). Dysbiosis, imbalance in function or structure of gut microbiota, has been associated with a variety of diseases, such as inflammatory bowel disease, obesity, colitis, and colorectal cancer (11-15).

**Figure 1:**
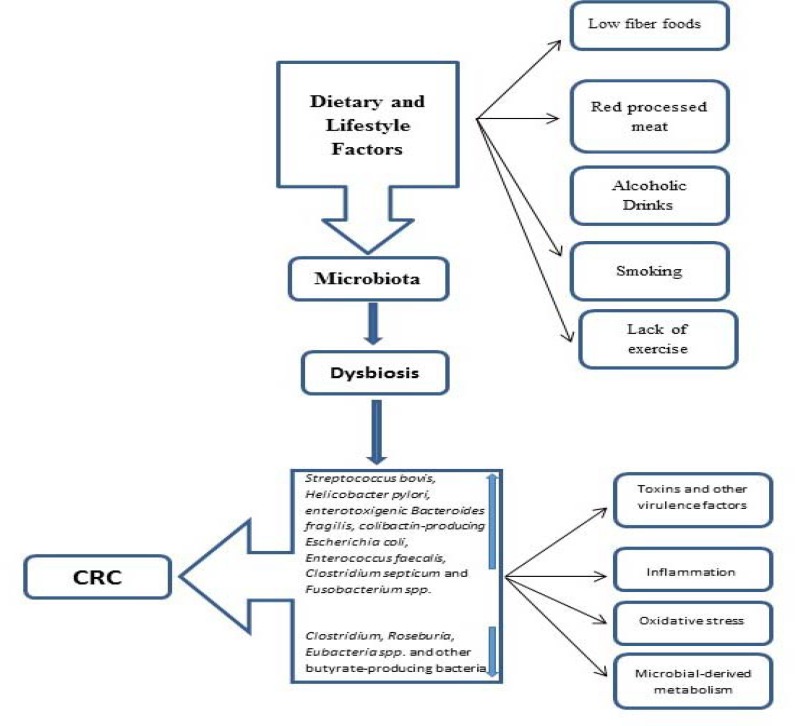
The linked of environment factors influenced gastrointestinal microbiota and promote colorectal cancer via various mechanisms

Although the gut microbiota have long been considered commensal residents in the gut, recent studies have demonstrated that microbiota may contribute to CRC pre-carcinogenesis (16). In certain conditions the intestinal microbiota may be linked to an increase in the risk of carcinogenesis and promote tumor growth via various mechanisms (Figure 1) (9). The relationship between cancer and microorganisms has been demonstrated in some organs, with the most well-known example which is the relationship between *Helicobacter pylori* and gastric cancer and mucosa-associated lymphoid tissue lymphoma or *papillomavirus* and cervical cancer (17, 18). Hence, there is much interest in understanding the composition of the gut microbiota in CRC patients in comparison to the healthy population as this knowledge may help develop new therapeutic methods for microbiota manipulation in benefit of the hosts health and disease prevention strategies. 

The aim of this review is to present the possible links between dysbiosis in the gut microbiota and colorectal cancer, and discuss the potential role of gut microbiota in the pathogenesis of colorectal cancer. 

## Methods

Searches were performed in PubMed, Medline, Google scholar, for articles published in English, and other bibliographic references and appropriate sources such as SID and Magiran for Persian-language journals from 2000 to December 2017 using the following keywords alone or in combination: “anaerobic,” “ microbiota,” “pathogenesis,” “colorectal cancer,” “microbiome,” “microbiota,” and “dysbiosis.” However, according to our explorer, no Persian-language papers were found. In total, 141 studies were published regarding the microbiota composition in colorectal cancer and based on the study scopes, we categorized all the papers into four major categories including gut microbiota colonization, frequency of gut microbiota, microbiota influence, and inflammatory Pathways.

**Gut microbiota in colorectal cancer patients**

Several studies have shown that numerous bacterial species appear to be associated with the pathogenesis of CRC and recent studies have provided a mechanism for the participation of gut microbiota in the progress of CRC (14, 19-22). Some bacterial species like *Clostridium septicum*, *Enterococcus faecalis*, *Streptococcus bovis*, *Bacteroides fragilis,* *Helicobacter pylori*, *Escherichia coli* and *Fusobacterium* spp. have been detected and supposed to play a role in colorectal pathogenesis (19-24). 

For example, *Streptococcus gallolyticus* (In the past *Streptococcus bovis*) is reported in nearly 20–50% and 5% of colon tumors and normal colon respectively. In CRC patients *Ruminococcus bromii,* *Clostridium clostridioforme* and* Bifidobacterium longum *have low prevalence compared to normal population (23). Furthermore, in different studies a notably increase number of the *Bacteroides/Prevotella* and *Fusobacterium nucleatum* population is described in CRC population (24). 

 **Frequency and pathogenesis of gut microbiota**

Recent investigations have confirmed strong relations between the development of colorectal cancer and gut microbiota (25-58). According to global investigations, the most predominant species of the adult health intestinal microbiota are *Bacteroidetes* and *Firmicutes* followed by *Actinobacteria*, *Proteobacteria*, and *Verrucomicrobia* but the composition and frequency of following microbiota changed in CRC patients (25). Intestinal microbiota can contribute to carcinogenesis through production of secondary metabolites, such as reactive oxygen intermediates that caused DNA damage, or direct effects on cell transformation through the production of genotoxin. Different bacterial species such as *Bacteroides fragilis*, *Clostridium septicum*, *Enterococcus faecalis*, *H. pylori*, *Streptococcus bovis*, *Escherichia coli*, and* Fusobacterium *spp. are supposed to play a role in colorectal carcinogenesis (table 1and 2) (26-58). Meanwhile the mechanisms of some of these bacteria were partly recognized. 

**Table 1:** Summary of increased gut microbiota variations in fecal sample of colorectal cancer patients

Reference	Sample type	Increased bacteria	Method
Sinha et al. 2016(51)	lyophilized feces	*Fusobacterium and Porphyromonas*	16S rRNA gene pyrosequencing
Flemer et al. 2016(52)	Fecal and mucosal samples	*Bacteroidetes and Prevotella*	16S rRNA amplicon sequencing
Wong et al. 2016(53)	Fecal samples	*F. nucleatum*	qPCR
Liang et al. 2016(54)	Fecal samples	*F. nucleatum*	qPCR
Suehiro et al. 2016(55)	Fecal samples	*F. nucleatum*	droplet digital PCR
Kasai et al. 2016(58)	Fecal samples	*Actinomyces odontolyticus, Bacteroides fragiles, Clostridium nexile, Fusobacterium varium, Haemophilus parainfluenzae, Prevotella stercorea, Streptococcus gordonii, and Veillonella dispar*	T-RFLP and NGS
Fukugaiti et al. 2015(45)	Fecal samples	*F. nucleatum *and* C. difficile*	qRT-PCR
Chen et al. 2013(68)	feces	*Enterococcus and Streptococcus*	Pyrosequencing based on 16S ribosomal RNA.
Wu et al. 2013 (69)	Feces	*enriched Bacteroides,* *overabundance of Fusobacterium and Campylobacter*	pyrosequencing of the 16S rRNA gene V3 region
Sobhani et al. 2011 (23)	Fecal samples	*Bacteroides/Prevotella*	Pyrosequencing and qRT-PCR
Ahn et al. 2013 (70)	Feces	*Atopobium/Porphyromonas and Fusobacterium*	Pyrosequencing and Real-time PCR
Balamurugan et al. 2008 (34)	Feces	*Enterococcus Faecalis*	Real-time PCR

*F. nucleatum: Fusobacterium nucleatum; E.faecalis:* *Enterococcus faecalis;* ETBF*:* *Enterotoxigenic Bacteroides fragilis;* qPCR: quantitative polymerase chain reaction; qRT-PCR: quantitative Real-time PCR; FQ-PCR: Fluorescent quantitative polymerase chain reaction; T-RFLP: terminal restriction fragment length polymorphism; NGS: next-generation sequencing

In different studies the prevalence of *S. bovis/gallolyticus *and* C. septicum *in CRC patients was reported from 33% to 100% and up to 40% respectively (33-36). In their meta-analysis study, Boleij et al confirmed the relationship between *S. bovis/gallolyticus *and *C. septicum *infections and CRC (59). *C. septicum* normally grows in soil and does not represent part of the normal bowel flora but there is no clear mechanism to explain the frequent association between *C.septicum* infection and colon cancer (31,33). *S. bovis/gallolyticus* bacteria were found in 2.5-15% of the normal population but significantly increased in CRC patients (36). *S. bovis/gallolyticus* could colonize and grow in colorectum tissues via collagen-binding and histone-like protein A to collagen I, IV, fibronectin, fibrinogen in colon tissues (35). The activity of present microbiota causes severe inflammatory response by inducing inflammatory and angiogenic cytokines in colorectum tissues and leading to the development or proliferation of colorectal cancer (3).

**Table 2:** Summary of increased gut microbiota variations in biopsy sample of colorectal cancer patients

Reference	Sample type	Increased bacteria	Method
Mima et al. 2016(49)	Tumor tissue samples	*F. nucleatum*	quantitative PCR assay
Wei et al. 2016(50)	Tumor tissue samples	*F. nucleatum and Bacteroides fragilis*	16S rRNA gene pyrosequencing
Li et al. 2016(56)	Tumor tissue samples	*F. nucleatum*	FQ-PCR
Zhou et al. 2016(57)	Tumor tissue samples	*Fusobacterium spp., E.faecalis, ETBF*	Real-time PCR
Burns et al. 2015(46)	Tumor tissue samples	*Fusobacterium *and* Providencia*	qPCR and 16S rRNA gene pyrosequencing
Gao et al. 2015(47)	Tumor tissue samples	*Firmicutes *and *Fusobacteria*	16S rRNA gene pyrosequencing
Mira-Pascual et al. 2015(48)	mucosal and fecal samples	*F. nucleatum and Enterobacteriaceae*	qPCR and 16S ribosomal RNA gene pyrosequencing
Viljoen et al. 2015(75)	CRC tissues	*fusobacterium spp., enterotoxigenic* *Bacteroides fragilis (ETBF)*	Real-time PCR
Tahara et al. 2014 (76)	CRC tissues	*F. nucleatum and Pan-fusobacterium*	Real-time PCR
Geng et al. 2013(77)	Tumor/matching normal tissue of Chinese CRC patients	*Fusobacterium spp., Roseburia*	pyrosequencing-based molecular monitoring of bacterial 16S rRNA gene
Warren et al. 2013(78)	CRC/matching normal tissues	*Fusobacterium, Leptotrichia and Campylobacter*	16S rRNA gene pyrosequencing
Castellarin et al. 2012(14)	Tumor/matching normal tissues	*Fusobacterium nucleatum*	RNA sequencing
Kostic et al. 2012 (65)	Tumor/matching normal tissues	*Fusobacterium*	whole genome sequences and confirmed by quantitative PCR and 16S rDNA sequence
Marchesi et al. 2011 (20)	Tumor/matching normal tissues	Fusobacterium	rRNA sequencing

 In a study by Sobhani et al, 179 subjects including 60 colorectal cancer and 119 healthy individuals underwent colonoscopy and the results showed higher levels of *Bacteroides/Prevotella* in patients with colorectal cancer (23). *Entrotoxigenic, Bacteroides fragilis *increased in fecal samples of CRC patients. *B. fragilis* degraded the E-cadherin protein and activated nuclear beta-catenin signaling and induces c-Myc expression and cellular proliferation (30, 60).

In a study by Gao *et al*. no significant difference was observed between proximal and distal colon microbiota in 30 healthy compare to 31 cancer patients; nevertheless, in colorectal cancer patients, *Firmicutes* and *Fusobacteria,* *Lactococcus* and *Fusobacterium* were more prevalent and *Proteobacteria,* *Pseudomonas* and *Escherichia*–*Shigella* were less frequent in tissues samples compared to control group (47).

Several studies showed higher prevalence of *F. nucleatum* in CRC tissue compare to a matched normal tissue (14, 19, and 24). *F. nucleatum* is showed as a probable candidate for CRC predisposition (61-66). *F. nucleatum* adheres to colonic epithelial cells through its FadA adhesion. FadA binds to E-cadherin, activates β-catenin signaling, and differentially regulates the inflammatory and oncogenic responses (41). Fap2 protein of *F. nucleatum* can stimulates CRC expansion by inhibition of the antitumor immune cell activity via TIGIT (67).

*Enterococcus faecalis (E. faecalis*), a commensal microorganism in the intestinal tract, has been repeatedly found in colorectal cancer patients (34, 68). *E*.*faecalis* has recently been considered as a human pathogen (68). Balamurugan et al. had reported statistically significant higher levels of *E. faecalis* from the feces of patients with CRC compared to healthy volunteers (34). These bacteria can produce reactive oxygen and nitrogen species (RONS) that directly lead to DNA break, point mutation and chromosomal instability. These functions demonstrated this common colonic commensal has rendered an organism with the potential to contribute to oncogenic transformation in the colon (68).

Controversial result have been reported regarding the role of *H. pylori *in CRC. Zumkeller et al., in their meta-analysis study, reported a 1.4 time increased risk of CRC in patients with a *H. pylori *infection around the world (39). Guo *et al,* in a meta-analysis study of 7679 Asian patients, recommended a carcinogenic role of *H. pylori *at a primary phase of carcinogenesis (69). Bacterial cytotoxin-associated gene A (CagA) and vacuolating cytotoxin A (VacA) are encoded in some *H. pylori* strains and induce the activation of inflammation pathways (70). There is also another hypothesis that direct and indirect production of RONS by some strains could participate in tumorigenesis in the colon (71).

While *E. coli *is a commensal microbe of the human gut, several surveys have verified a strong association between mucosa-adherent *E. coli *and CRC (42-44). In 2004 Martin et al. reported that more than 70% of mucosa samples of CRC patients were inhabited by *E.coli* (42). Majority of *E. coli* isolated from CRC patients harbors the *pks* genomic island that is responsible for the synthesis of colibactin. Colibactin is another bacterial-derived genotoxin that can interference with the cell cycle and promote proliferation of epithelial cells via DNA damage, mutation and genomic instability, subsequently and, tumor growth (44). 

**Colon cancer and inflammatory pathways**

As mentioned, increased gut microbiota release inflammatory agents via the inflammatory pathway and therefore promote the change of normal cells to cancerous cells. On the other hand, intestinal inflammation as observed in inflammatory bowel disease (IBD) is a risk factor for the development of CRC (72). Increasing evidence suggests that inflammation-associated pathways also contribute to CRC development in the absence of clinically overt intestinal inflammation. Thus, signaling pathways with central roles in myeloid and lymphoid cells, such as those associated with signal transducer and activator of transcription 3 (STAT3) and nuclear factor (NF)-κB, are also active in the transformed intestinal epithelium and promote tumor development (73, 74). 

A study by Wang et al. has recently shown a critical role of the microbiota, and its TLR-dependent recognition in intestinal tumor progress in human and rodents (75). Intestinal microbiota are also known to be involved in the initiation and development of colorectal cancer, which is a risk factor for inflammatory bowel disease. 

The investigations conﬁrmed profound modifications in the gut microbiota before or during the progression of colorectal cancer (9). Intestinal microbiota-dependent nutritional or lifestyle intermediation beside colorectal carcinoma deserve additional research. The result of different studies advocate that fecal microbiome-based approaches might be valuable for prompt diagnosis and treatment of colorectal cancer. 

## Conclusion

According to the presented studies, more prevalent gut microbiota variations in the fecal and biopsy samples of CRC patients were *Fusobacterium*, *Porphyromonas*, *Bacteroidetes* and *Prevotella*. (Table 1, 2). However, it seems that there is no difference between bacteria variation in developed and developing countries. Therefore, the strong association between the gut microbiota and CRC is evident, but several questions remain unanswered. As previously declared, the gut microbiota acts as a key role in the development of CRC through numerous mechanisms, comprising genotoxin, metabolism and inflammation. Thus, studies have provided supportive data that modifications in gut microbiota structure could induce a host immune response and plays a critical part in intestinal epigenic mechanisms of the host.

The studies that are discussed in this review did not highlight the classification of tumors according to their molecular phenotype and it is not clear why some adenomas growth to malignancy, while others are stable or even regress. According to investigations, a greater abundance of *Fusobacterium* was detected in cancer tissues than in normal tissues. Thus, the increased abundance of *Fusobacterium* could be linked with high risk of CRC.

Therefore, we recommended future studies should consider the heterogeneity of CRC tumors by focusing on microbiota imbalances in relation to molecular pathways involved in colorectal carcinogenesis. Also performing such studies may be useful to explore links between pathological features of adenomas and type of cytotoxic microbiota. On the other hand, development of research techniques are expected to provide important evidence concerning healthy and dysbiotic microbiota conformation. In conclusion, the role of the gut microbiota in the pathogenesis of CRC is clear and perhaps represents new techniques for better therapeutic management of patients with CRC.

## Conflict of interests

The authors declare that they have no conflict of interest.

## References

[1] Haggar FA, Boushey RP (2009). Colorectal cancer epidemiology: incidence, mortality, survival, and risk factors. Clin Colon Rectal Surg.

[2] Pourhoseingholi MA, Zali MR (2012). Colorectal cancer screening: Time for action in Iran. World J Gastrointest Oncol.

[3] Zahir MN, Azhar EM, Rafiq S, Ghias K, Shabbir-Moosajee M (2014). Clinical features and outcome of sporadic colorectal carcinoma in young patients: a cross-sectional analysis from a developing country. ISRN Oncol.

[4] Carethers JM, Jung BH (2015). Genetics and Genetic Biomarkers in Sporadic Colorectal Cancer. Gastroenterology.

[5] Watson A J, Collins PD (2011). Colon cancer: a civilization disorder. Dig Dis.

[6] Azimirad M, Rostami-Nejad M, Rostami K, Naji T, Zali MR (2015). The Susceptibility of Celiac Disease Intestinal Microbiota to Clostridium difficile Infection. Am J Gastroenterol.

[7] Rostami Nejad M, Ishaq S, Al Dulaimi D, Zali MR, Rostami K (2015). The role of infectious mediators and gut microbiome in the pathogenesis of celiac disease. Arch Iran Med.

[8] Phillips ML (2009). Gut reaction: environmental effects on the human microbiota. Environ Health Perspect.

[9] Gagnière J, Raisch J, Veziant J, Barnich N, Bonnet R, Buc E (2016). Gut microbiota imbalance and colorectal cancer. World J Gastroenterol.

[10] den Besten G, van Eunen K, Groen AK, Venema K, Reijngoud DJ, Bakker BM (2013). The role of short-chain fatty acids in the interplay between diet, gut microbiota,and host energy metabolism. J Lipid Res.

[11] Lepage P, Leclerc MC, Joossens M, Mondot S, Blottiere HM, Raes J (2012). A metagenomic insight into our gut's microbiome. Gut.

[12] Lakhan, Shaheen E, Kirchgessner, Annette (2010). Gut inflammation in chronic fatigue syndrome. Nut Metabol.

[13] Turnbaugh PJ, Ley RE, Mahowald MA, Magrini V, Mardis ER, Gordon JI (2006). An obesity-associated gut microbiome with increased capacity for energy harvest. Nature.

[14] Castellarin M, Warren RL, Freeman JD, Dreolini L, Krzywinski M, Strauss J (2012). Fusobacterium nucleatum infection is prevalent in human colorectal carcinoma. Genome Res.

[15] Mazmanian SK (2008). Capsular polysaccharides of symbiotic bacteria modulate immune responses during experimental colitis. J Pediatr Gastroenterol Nutr.

[16] Ferlay J, Soerjomataram I, Dikshit R, Eser S, Mathers C, Rebelo M (2015). Cancer incidence and mortality worldwide: sources, methods and major patterns in GLOBOCAN 2012. Int J Cancer.

[17] Burd EM (2003). Human papillomavirus and cervical cancer. Clin Microbiol Rev.

[18] Park JB, Koo JS (2014). Helicobacter pylori infection in gastric mucosa-associated lymphoid tissue lymphoma. World J Gastroenterol.

[19] Kostic AD, Chun E, Robertson L, Glickman JN, Gallini CA, Michaud M (2013). Fusobacterium nucleatum potentiates intestinal tumorigenesis and modulates the tumor-immune microenvironment. Cell Host Microbe.

[20] Marchesi JR, Dutilh BE, Hall N, Peters WH, Roelofs R, Boleij A (2011). Towards the human colorectal cancer microbiome. PLoS One.

[21] Srikanth CV, McCormick BA (2008). Interactions of the intestinal epithelium with the pathogen and the indigenous microbiota: a three-way crosstalk. Interdiscip Perspect Infect Dis.

[22] Wei H, Dong L, Wang T, Zhang M, Hua W, Zhang C (2010). Structural shifts of gut microbiota as surrogate endpoints for monitoring host health changes induced by carcinogen exposure. FEMS Microbiol Ecol.

[23] Sobhani I, Tap J, Roudot-Thoraval F, Roperch JP, Letulle S, Langella P (2011). Microbial dysbiosis in colorectal cancer (CRC) patients. PLoS One.

[24] Ray K (2011). Colorectal cancer: Fusobacterium nucleatum found in colon cancer tissue--could an infection cause colorectal cancer?. Nat Rev Gastroenterol Hepatol.

[25] Bäckhed F, Ley RE, Sonnenburg JL, Peterson DA, Gordon JI (2005). Host-bacterial mutualism in the human intestine. Science.

[26] Tjalsma H, Boleij A, Marchesi JR, Dutilh BE (2012). A bacterial driver passenger model for colorectal cancer: beyond the usual suspects. Nat Rev Microbiol.

[27] Wang T, Cai G, Qiu Y, Fei N, Zhang M, Pang X (2012). Structural segregation of gut microbiota between colorectal cancer patients and healthy volunteers. ISME J.

[28] Housseau F, Sears CL (2010). Enterotoxigenic Bacteroides fragilis (ETBF)-mediated colitis in Min (Apc+/-) mice: a human commensal-based murine model of colon carcinogenesis. Cell Cycle.

[29] Toprak NU, Yagci A, Gulluoglu BM, Akin ML, Demirkalem P, Celenk T (2006). A possible role of Bacteroides fragilis enterotoxin in the aetiology of colorectal cancer. Clin Microbiol Infect.

[30] Wu S, Morin PJ, Maouyo D, Sears CL (2003). Bacteroides fragilis enterotoxin induces c-Myc expression and cellular proliferation. Gastroenterology.

[31] Chew SS, Lubowski DZ (2001). Clostridium septicum and malignancy. ANZ J Surg.

[32] Hermsen JL, Schurr MJ, Kudsk KA, Faucher LD (2008). Phenotyping Clostridium septicum infection: a surgeon’s infectious disease. J Surg Res.

[33] Mirza NN, McCloud JM, Cheetham MJ (2009). Clostridium septicum sepsis and colorectal cancer - a reminder. World J Surg Oncol.

[34] Balamurugan R, Rajendiran E, George S, Samuel GV, Ramakrishna BS (2008). Real-time polymerase chain reaction quantification of specific butyrate-producing bacteria, Desulfovibrio and Enterococcus faecalis in the feces of patients with colorectal cancer. J Gastroenterol Hepatol.

[35] Abdulamir AS, Hafidh RR, Abu Bakar F (2011). The association of Streptococcus bovis/gallolyticus with colorectal tumors: the nature and the underlying mechanisms of its etiological role. J Exp Clin Cancer Res.

[36] Klein RS, Recco RA, Catalano MT, Edberg SC, Casey JI, Steigbigel NH (1977). Association of Streptococcus bovis with carcinoma of the colon. N Engl J Med.

[37] Grahn N, Hmani-Aifa M, Fransén K, Söderkvist P, Monstein HJ (2005). Molecular identification of Helicobacter DNA present in human colorectal adenocarcinomas by 16S rDNA PCR amplification and pyrosequencing analysis. J Med Microbiol.

[38] Jones M, Helliwell P, Pritchard C, Tharakan J, Mathew J (2007). Helicobacter pylori in colorectal neoplasms: is there an aetiological relationship?. World J Surg Oncol.

[39] Zumkeller N, Brenner H, Zwahlen M, Rothenbacher D (2006). Helicobacter pylori infection and colorectal cancer risk: a metaanalysis. Helicobacter.

[40] McCoy AN, Araújo-Pérez F, Azcárate-Peril A, Yeh JJ, Sandler RS, Keku TO (2013). Fusobacterium is associated with colorectal adenomas. PLoS One.

[41] Rubinstein MR, Wang X, Liu W, Hao Y, Cai G, Han YW (2013). Fusobacterium nucleatum promotes colorectal carcinogenesis by modulating E-cadherin/β-catenin signaling via its FadA adhesin. Cell Host Microbe.

[42] Martin HM, Campbell BJ, Hart CA, Mpofu C, Nayar M, Singh R (2004). Enhanced Escherichia coli adherence and invasion in Crohn’s disease and colon cancer. Gastroenterology.

[43] Swidsinski A, Khilkin M, Kerjaschki D, Schreiber S, Ortner M, Weber J (1998). Association between intraepithelial Escherichia coli and colorectal cancer. Gastroenterology.

[44] Arthur JC, Perez-Chanona E, Mühlbauer M, Tomkovich S, Uronis JM, Fan TJ (2012). Intestinal inflammation targets cancer-inducing activity of the microbiota. Science.

[45] Fukugaiti MH, Ignacio A, Fernandes MR, Ribeiro Júnior U, Nakano V, Avila-Campos (2015). occurrence of Fusobacterium nucleatum and Clostridium difficile in the intestinal microbiota of colorectal carcinoma patients. Braz J Microbiol.

[46] Burns MB, Lynch J, Starr TK, Knights D, Blekhman R (2015). Virulence genes are a signature of the microbiome in the colorectal tumor microenvironment. Genome Med.

[47] Gao Z, Guo B, Gao R, Zhu Q, Qin H (2015). Microbiota disbiosis is associated with colorectal cancer. Front Microbiol.

[48] Mira-Pascual L, Cabrera-Rubio R, Ocon S, Costales P, Parra A, Suarez A (2015). Microbial mucosal colonic shifts associated with the development of colorectal cancer reveal the presence of different bacterial and archaeal biomarkers. J Gastroenterol.

[49] Mima K, Cao Y, Chan AT, Qian ZR, Nowak JA, Masugi Y (2016). Fusobacterium nucleatum in Colorectal Carcinoma Tissue According to Tumor Location. Clin Transl Gastroenterol.

[50] Wei Z, Cao S, Liu S, Yao Z, Sun T, Li Y (2016). Could gut microbiota serve as prognostic biomarker associated with colorectal cancer patients' survival? A pilot study on relevant mechanism. Oncotarget.

[51] Sinha R, Ahn J, Sampson JN, Shi J, Yu G, Xiong X (2016). Fecal Microbiota, Fecal Metabolome, and Colorectal Cancer Interrelations. PLoS One.

[52] Flemer B, Lynch DB, Brown JM, Jeffery IB, Ryan FJ, Claesson MJ (2017). Tumour-associated and non-tumour-associated microbiota in colorectal cancer. Gut.

[53] Wong SH, Kwong TNY, Chow TC, Luk AKC, Dai RZW, Nakatsu G (2017). Quantitation of faecal Fusobacterium improves faecal immunochemical test in detecting advanced colorectal neoplasia. Gut.

[54] Liang Q, Chiu J, Chen Y, Huang Y, Higashimori A, Fang J (2017). Fecal Bacteria Act as Novel Biomarkers for Noninvasive Diagnosis of Colorectal Cancer. Clin Cancer Res.

[55] Suehiro Y, Sakai K, Nishioka M, Hashimoto S, Takami T, Higaki S (2017). Highly sensitive stool DNA testing of Fusobacterium nucleatum as a marker for detection of colorectal tumours in a Japanese population. Ann Clin Biochem.

[56] Li YY, Ge QX, Cao J, Zhou YJ, Du YL, Shen B (2016). Association of Fusobacterium nucleatum infection with colorectal cancer in Chinese patients. World J Gastroenterol.

[57] Zhou Y, He H, Xu H, Li Y, Li Z, Du Y (2016). Association of oncogenic bacteria with colorectal cancer in South China. Oncotarget.

[58] Kasai C, Sugimoto K, Moritani I, Tanaka J, Oya Y, Inoue H (2016). Comparison of human gut microbiota in control subjects and patients with colorectal carcinoma in adenoma: Terminal restriction fragment length polymorphism and next-generation sequencing analyses. Oncol Rep.

[59] Boleij A, van Gelder MM, Swinkels DW, Tjalsma H (2011). Clinical Importance of Streptococcus gallolyticus infection among colorectal cancer patients: systematic review and meta-analysis. Clin Infect Dis.

[60] Boleij A, Hechenbleikner EM, Goodwin AC, Badani R, Stein EM, Lazarev MG (2015). The Bacteroides fragilis toxin gene is prevalent in the colon mucosa of colorectal cancer patients. Clin Infect Dis.

[61] Kostic AD, Gevers D, Pedamallu CS, Michaud M, Duke F, Earl AM (2012). Genomic analysis identifies association of Fusobacterium with colorectal carcinoma. Genome Res.

[62] Ahn J, Sinha R, Pei Z, Dominianni C, Wu J, Shi J (2013). Human gut microbiome and risk for colorectal cancer. J Natl Cancer Inst.

[63] Viljoen KS, Dakshinamurthy A, Goldberg P, Blackburn JM (2015). Quantitative profiling of colorectal cancer-associated bacteria reveals associations between fusobacterium spp enterotoxigenic Bacteroides fragilis (ETBF) and clinicopathological features of colorectal cancer. PLoS One.

[64] Tahara T, Yamamoto E, Suzuki H, Maruyama R, Chung W, Garriga J (2014). Fusobacterium in colonic flora and molecular features of colorectal carcinoma. Cancer Res.

[65] Geng J, Fan H, Tang X, Zhai H, Zhang Z (2013). Diversified pattern of the human colorectal cancer microbiome. Gut Pathog.

[66] Warren RL, Freeman DJ, Pleasance S, Watson P, Moore RA, Cochrane K (2013). Co-occurrence of anaerobic bacteria in colorectal carcinomas. Microbiome.

[67] Gur C, Ibrahim Y, Isaacson B, Yamin R, Abed J, Gamliel M (2015). Binding of the Fap2 protein of Fusobacterium nucleatum to human inhibitory receptor TIGIT protects tumors from immune cell attack. Immunity.

[68] Pillar CM, Gilmore MS (2004). Enterococcal virulence--pathogenicity island of E Faecalis. Front Biosci.

[69] Guo Y, Li HY (2014). Association between Helicobacter pylori infection and colorectal neoplasm risk: a meta-analysis based on East Asian population. J Cancer Res Ther.

[70] Higashi H, Tsutsumi R, Fujita A, Yamazaki S, Asaka M, Azuma T (2002). Biological activity of the Helicobacter pylori virulence factor CagA is determined by variation in the tyrosine phosphorylation sites. Proc Natl Acad Sci USA.

[71] Handa O, Naito Y, Yoshikawa T (2010). Helicobacter pylori: a ROS-inducing bacterial species in the stomach. Inflamm Res.

[72] Terzić J, Grivennikov S, Karin E, Karin M (2010). Inflammation and colon cancer. Gastroenterology.

[73] Bollrath J, Phesse TJ, von Burstin VA, Putoczki T, Bennecke M, Bateman T (2009). gp130-mediated Stat3 activation in enterocytes regulates cell survival and cell-cycle progression during colitis-associated tumorigenesis. Cancer Cell.

[74] Greten FR, Eckmann L, Greten TF, Park JM, Li ZW, Egan LJ (2004). IKKbeta links inflammation and tumorigenesis in a mouse model of colitis-associated cancer. Cell.

[75] Wang EL, Qian ZR, Nakasono M, Tanahashi T, Yoshimoto K, Bando Y (2010). High expression of Toll-like receptor 4/myeloid differentiation factor 88 signals correlates with poor prognosis in colorectal cancer. Br J Cancer.

